# Proximal Aortic Dilatation and Pulmonary Valve Replacement in Patients with Repaired Tetralogy of Fallot: Is There a Relationship? A Cardiac Magnetic Resonance Imaging Study

**DOI:** 10.3390/jcm10225296

**Published:** 2021-11-15

**Authors:** Ahmed Farghal A. Mohammed, Michael Frick, Gunter Kerst, Nima Hatam, Mohamed-Adel F. Elgamal, Karam M. Essa, Hedwig H. Hövels-Gürich, Jaime F. Vazquez-Jimenez, Rashad Zayat

**Affiliations:** 1 Department of Pediatric Cardiac Surgery, RWTH University Hospital Aachen, Faculty of Medicine, Pauwelsstr. 30, 52074 Aachen, Germany; 2 Department of Cardiothoracic Surgery, Qena University Hospitals, Faculty of Medicine, South Valley University, Qena 83511, Egypt; me.karam@ymail.com; 3 Department of Thoracic and Cardiovascular Surgery, RWTH University Hospital Aachen, Faculty of Medicine, Pauwelsstr. 30, 52074 Aachen, Germany; nhatam@ukaachen.de (N.H.); rzayat@ukaachen.de (R.Z.); 4 Department of Cardiology, RWTH University Hospital Aachen, Faculty of Medicine, Pauwelsstr. 30, 52070 Aachen, Germany; mfrick@ukaachen.de; 5 Department of Pediatric Cardiology, RWTH University Hospital Aachen, Faculty of Medicine, Pauwelsstr. 30, 52074 Aachen, Germany; gkerst@ukaachen.de (G.K.); hhoevels-guerich@ukaachen.de (H.H.H.-G.); 6 Cardiovascular Surgery, Mansoura University, Mansoura 35516, Egypt; adelelgamal@live.com

**Keywords:** pulmonary valve replacement, tetralogy of Fallot, aortopathy, aortic aneurysm, adult congenital heart disease, comorbidities, CMR, magnetic resonance imaging

## Abstract

Aortopathy is a known complication whose incidence is growing within the population of tetralogy of Fallot (TOF) patients. Its pathology and relationship with other comorbidities remain unclear. This study was designed to determine the prevalence and predictors of proximal aortic dilatation after TOF repair. We retrospectively investigated all patients who underwent follow-up cardiac magnetic resonance imaging (CMR; at least 4 years after TOF repair) between March 2004 and December 2019. The dimensions at the ascending aorta (AAo) and sinus of Valsalva (SoV) levels were measured. Aortic dilatation was defined as an internal aortic diameter that was >2 standard deviation of the previously published normal values. We included 77 patients (mean age 28.9 ± 10.5 years, 41.5% female, mean follow-up of 24.5 ± 8.1 years). AAo and SoV were dilated in 19 (24.6%) and 43 (55.8%) patients, respectively. Patients with dilated AAo and SoV were older during the corrective surgery (*p* < 0.001 and *p* = 0.004, respectively) and during CMR (*p* = 0.002 and 0.024, respectively) than patients without AAo and SoV dilatation. Patients of the dilated AAo group were more likely to have prior palliative shunt (*p* = 0.008), longer shunt duration (*p* = 0.005), and a higher degree of aortic valve regurgitation (AR) fraction (*p* < 0.001) and to undergo pulmonary (PVR) and/or aortic valve replacement (*p* < 0.001 and *p* = 0.013, respectively). PVR (*p* = 0.048, odds ratio = 6.413, and 95% CI = 1.013–40.619) and higher AR fraction (*p* = 0.031, odds ratio = 1.194, and 95% CI = 1.017–1.403) were independent predictors for AAo dilatation. Aortopathy is a common progressive complication that may require reintervention and lifelong follow-up. Our study shows that proximal aortic dilatation may be attributed to factors that increase the volume overload across the proximal aorta, including late corrective surgery and palliative shunt. We also found that PVR and higher AR fraction are independent predictors of AAo dilatation.

## 1. Introduction

Tetralogy of Fallot (TOF) is the most common cyanotic congenital heart disease. Since the first successful correction by Lillehei et al. in 1955 [1], marked improvements have been made in strategies for its management, resulting in excellent survival rates, worldwide, thereby presenting a constantly growing adult population with repaired TOF (rTOF) [2,3]. This cohort of patients requires lifelong routine follow-up, where reintervention rates are as high as 44%. Such reintervention most commonly manages progressive pulmonary valve regurgitation (PR), often requiring pulmonary valve replacement (PVR) [4]. Proximal aortic dilatation is another known comorbidity that may show a progressive nature in some patients and thus possibly require surgical intervention. Despite the progressive nature of aortopathy, thoracic aortic dissection (TAD) is rarely reported [5]. However, the need for aortic valve surgery due to progressive aortic valve regurgitation (AR) is not uncommon [6]. Aortopathy is gaining increasing interest, but, unfortunately, clear data on the definition of aortic dilatation, the associated factors and predictors of aortic dilatation, and subsequent complications are lacking. Moreover, its relationship with other comorbidities that might require coincident reintervention needs to be explored.

This study aimed to examine the proximal aorta diameters at the level of the sinus of Valsalva (SoV) and ascending aorta (AAo) using cardiac magnetic resonance imaging (CMR) in an adult cohort of patients with rTOF and to analyze the possible associated factors and predictors of proximal aortic dilatation and its possible relation with other comorbidities, which would assist in the development of future therapeutic interventions and strategies with better patient outcomes.

## 2. Materials and Methods

### 2.1. Study Population

All adult patients (aged ≥ 18 years) with rTOF who underwent follow-up CMR (at least 4 years after corrective surgery) between March 2004 and December 2019 at our center were retrospectively investigated. Exclusion criteria were diagnosis with pulmonary atresia (PA), double outlet right ventricle (of TOF subtype), aortic valve endocarditis, aortic valve congenital abnormality, and/or connective tissue disorder.

### 2.2. Data Collection

Anatomical and surgical characteristics, including sidedness of the aortic arch, number and type of previous palliative shunts, duration of the palliative shunt, age at TOF corrective surgery, and history of residual ventricular septal defect (VSD), data regarding medical therapy, and surgical interventiondetails as indicated by the current CMR study (e.g., PVR, aortic valve replacement (AVR) or repair and AAo replacement) were collected and analyzed.

### 2.3. Cardiac Magnetic Resonance Imaging

All study patients were examined in the supine position using a 1.5 Tesla whole-body MR scanner (until 2008: Philips Intera; Philips Medical Systems, Best, The Netherlands; after 2008: Philips Achieva; Philips Medical Systems, Best, The Netherlands) equipped with a dedicated cardiac coil for parallel imaging and a vector electrocardiogram for cardiac synchronization. After survey scanning, a noncontrast, free-breathing navigator-gated, ECG-triggered (end-diastole) three-dimensional (3D) steady-state free precession (SSFP) sequence with T2 preparation and fat saturation was performed, covering the thorax from above the aortic arch to the diaphragm. Sequence parameters were slightly changed over the years. The typical parameters included a spatial resolution of 2 mm × 2 mm × 2 mm (reconstructed to 1 mm × 1 mm × 1 mm) and an acquisition duration of 130 ms per heartbeat. The typical net scan time was 2 to 3 minutes. Then, standard balanced SSFP cine imaging (retrospective gating; expiratory breath-hold) in three standard left ventricle (LV) long-axis views (2-, 4-, and 3-chamber views) and right ventricular outflow tract (RVOT) geometry in short-axis orientation with full ventricular coverage was performed. Next, a standard through-plane velocity-encoded phase-contrast mapping sequence (retrospective gating; end-expiratory breath-hold) was performed orthogonal to the ascending aorta at the level of the sino-tubular junction as well as in the proximal main pulmonary artery just above the pulmonary valve.

CMR data were analyzed offline on a dedicated MR workstation (Extended Workspace; Philips Healthcare, Best, The Netherlands). All measurements were performed by a cardiology consultant with >11 years of experience in CMR and Level 3 CMR Certification from the European Association of Cardiovascular Imaging (M.F). The measurement of aortic dimensions was repeated by A.F.A.M. a cardiothoracic surgery specialist with more than 10 years of experience under the supervision of M.F. Both readers are among the authors. LV and right ventricle (RV) volumes and ejection fraction were assessed from the short-axis stack according to the disc summation method (Simpson’s rule). In the flow-sensitive phase-contrast images, the cross-sectional areas of the ascending aorta and the pulmonary artery were semi-automatically tracked over the heart cycle, and the aortic and pulmonary valve regurgitation fractions were calculated.

**Figure 1:**
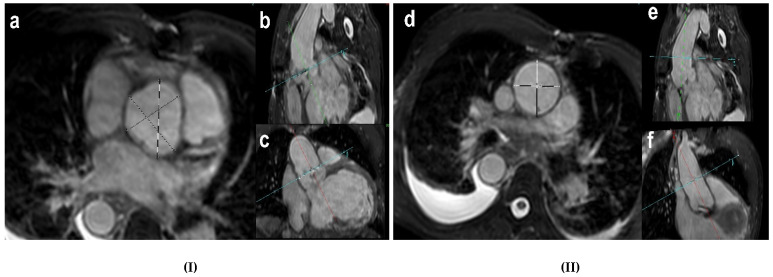
(**I**) (**a**). Transverse plane image at the level of SoV, end-diastolic capture, showing the measurement lines from cusp to cusp (black lines). (**b**). An oblique longitudinal LVOT cine image. (**c**). An oblique frontal LVOT cine image. (**II**) (**d**). Transverse plane image of AAo at the level of the left pulmonary artery, end-diastolic capture, showing the measurement lines (black lines). (**e**). An oblique longitudinal LVOT cine image. (**f**). An oblique frontal LVOT cine image. Green lines and red lines are parallel to blood flow and perpendicular to the blue line, which represents the equivalent level of measurements. AAo, ascending aorta; LVOT, left ventricular outflow tract; SoV, sinus of Valsalva.

Aortic diameters were derived from the end-diastolic 3D-SSFP images perpendicular to the vessel using a multiplanar reformation (MPR) tool (Figure 1). Diameters were measured at the SoV and the mid ascending aorta at the level of the pulmonary artery according to reference [7]. For the sinus of Valsalva, all three sinus-to-sinus diameters were measured, and the maximal diameter was used in this study. For the mid ascending aorta, the maximal diameter was derived from the cross-sectional MPR reformation and used in this study.

### 2.4. Definition of Proximal Aortic Dilatation

Proximal aortic dilatation was defined as an aortic inner luminal diameter indexed to body surface area (BSA) and adjusted for age and sex, where (mm/m^2^) > 2 standard deviations (SDs) of previously published normal CMR aortic dimensions by Burman et al. [8] at the level of the SoV and by Davis et al. [9] at the level of the AAo. 

### 2.5. Statistical Analysis

Continuous variables are expressed as the means ± SDs. Categorical variables are expressed as absolute numbers and percentages. Comparisons of continuous variables between patients with and without aortic dilatation were performed using independent Student *t*-tests or Man-Whitney-U-Test, depending on their distribution. Fisher’s exact test was used to compare categorical variables. Correlations between continuous variables were assessed using Spearman correlation coefficients. Univariate and Multivariate logistic regression were performed to identify predictors of Sov and AAo dilatation. Only those variables that were significant at *p* < 0.05 in the univariate logistic regression were included in a multivariable model. A stepwise option was used to determine independent predictors of the outcome variables. All statistical comparisons were two-sided and a *p* < 0.05 was considered to indicate significant differences. Analyses were performed using STATA (Release 16, StataCorp, College Station, TX, USA) and the Jamovi project (Version 2.2, https://www.jamovi.org (accessed on 21 May 2021)).

## 3. Results

Eighty-nine patients were screened in this study. Among them, 12 patients were excluded according to the pre-established exclusion criteria—8 patients with PA, 1 patient with aortic valve endocarditis, and 3 patients with incomplete CMR data. Therefore, 77 patients (41.5% female) with a mean age of 28.9 ± 10.5 years were included in this study. The mean follow-up duration was 24.5 ± 8.1 years after corrective surgery. Further, 20 patients (26%) were totally corrected during the first year of life, and 15 patients (19.5%) underwent palliative procedures before TOF repair, most commonly Blalock–Taussig shunt (BTS). A total of 23 patients (29.9%) had right-sided aortic arch, and 13 patients (16.8%) had residual VSD on the day of CMR examination.

### 3.1. Prevalence of Proximal Aortic Dilatation and Aortic Valve Regurgitation

AAo and SoV were dilated in 19 (24.6%) and 43 (55.8%) patients, respectively. Aortic diameters are listed in (Table 1). The inner diameter was ≥40 mm in 12 (15.58%) and 38 (49.35%) patients at the AAo and SoV levels, respectively. AAo was larger than SoV in 9% of the patients. AR was moderate (AR fraction ≥ 20%) to severe (AR fraction ≥ 40%) in 5 patients (6.5% of the entire study population and 26.3% and 11.5% of patients with dilated AAo and SoV, respectively), whereas the AR fraction was mild (AR fraction of 5–19%) in 24 patients (33% of the entire study population). 

### 3.2. Patients’ Characteristics

The patients’ basic characteristics are listed in (Table 2). Patients with dilated AAo and SoV were older during corrective surgery and during CMR examination and more likely to have a higher New York Heart Association (NYHA) functional class than patients without dilated AAo and SoV. Correction during the first year of life was associated with a lower likelihood of proximal aortic dilatation. Moreover, patients with AAo dilatation were more likely to undergo palliative shunt and have a longer shunt duration before corrective surgery. After TOF correction, the prevalence of residual VSD was higher in the AAo dilatation group. No differences in sex, aortic arch sidedness, or incidence of arterial hypertension were observed among the entire study population. 

### 3.3. Surgical Reinterventions

Most surgical interventions were indicated in the current CMR study. PVR was performed in 28 (36.3%) patients. The mean age at PVR was 36.5 ± 11.6 years, and the mean time after corrective surgery was 31.5 ± 10.1 years. One patient underwent PVR due to residual right ventricular outflow tract (RVOT) obstruction and pulmonary valve stenosis. The other 27 patients underwent reoperation due to significant PR. Four patients (21% of the patients in the AAo dilatation group) underwent aortic valve and/or AAo surgery due to severe AR or AAo aneurysm. Among these four patients, two underwent isolated aortic valve surgery, one underwent isolated supra-coronary AAo replacement, and one underwent a Bentall operation. Moreover, of the four patients, three were operated either with or after PVR. Surgeries were performed 8–36 years after primary correction, which was performed at ages 10–18 years. 

### 3.4. Factors Associated with Proximal Aortic Dilatation

In the correlation test, a higher AAo diameter indexed to BSA was positively correlated with age at TOF corrective surgery (Spearman’s rho = 0.490; *p* < 0.001), palliative shunt (Spearman’s rho = 0.327; *p* = 0.004), longer shunt duration (Spearman’s rho = 0.322; *p* = 0.004), and AR severity (Spearman’s rho = 0.598; *p* < 0.001). Furthermore, significant correlations were observed between older age at corrective surgery and AR severity (Spearman’s rho = 0.490; *p* < 0.001) and aortic valve (Spearman’s rho = 0.299, *p* = 0.009) and AAo (Spearman’s rho = 0.268, *p* = 0.02) surgeries. Moderate but highly significant correlations were observed between PVR and the incidence of AAo dilatation (Spearman’s rho = 0.444; *p* < 0.001) and between PVR and higher AAo diameter indexed to BSA (Spearman’s rho = 0.371; *p* < 0.001). PVR was correlated with trans-annular patch (TAP) implantation during primary corretion (Spearman’s rho = 0.32, *p* = 0.004).

Predictors of SoV and AAo dilatation according to univariate analysis are listed in (Table 3). In the multivariate analysis, PVR (*p* = 0.048; odds ratio [OR] = 6.413; 95% confidence interval [CI] = 1.013–40.619) and a higher AR fraction (*p* =0.031; OR = 1.194; and 95% CI = 1.017–1.403) were independent predictors of AAo dilatation. No predictor of SoV dilatation was found (Table 4).

## 4. Discussion

The extent of the prevalence of proximal aortic dilatation remains controversial, with values in reports ranging from 6% [10] to 87% [11]. This marked discrepancy may be attributed to (a) variation in the study cohort (age: adult vs. children and adolescents; inclusion vs. exclusion ofPA), (b) imaging technique, (c) aortic level being studied, and (d) cutoff value used to define dilatation. Unlike most studies, one study [12], in addition to this study, excluded patients with PA to eliminate the possible population bias resulting from differences in anatomical characteristics. Moreover, AR and aortic root dilatation are more prevalent in this subgroup of patients [13].

### 4.1. Aortic Dilatation: Definition, Prevalence, and Level of Measurement

The definition of aortic dilatation in patients with rTOF remains controversial. The cutoff value used to define dilatation can significantly affect the reported prevalence of aortic dilatation. For more accurate and elaborated determination of aortic dilatation, we decided to use aortic measurements indexed to the BSA, adjusted for age and sex and compared with the nomogram derived from previous CMR studies [8,9]. For the purpose of early detection and follow-up of patients with a tendency for aortic dilatation, we chose to use Z-score ≥ 2 as the cutoff for the definition of aortic dilatation. The detected prevalence in our study was close to that reported in other studies (i.e., a range of 21–50%) that used the same definition of dilatation [14,15]. A cutoff value of an observed-to-expected ratio of more than 1.5 as a definition of dilatation was used by some researchers, and the resulting prevalence was less than our reported prevalence (6.6–15%) considering differences in the aortic level under study [10,16,17]. 

The level of the examined aorta is another important issue in aortic measurement. In this study, we found that AAo had a larger diameter than SoV in 9% of the patients, which is close to the corresponding rate reported by C. Cruz et al. (12.8%) [12]. Saiki et al. reported a marked reduction in the wall elasticity of AAo in patients with rTOF [18]. Moreover, all previous case reports of aortic dissection showed AAo diameters of >55 mm with AAo being larger than SoV, and the described intimal tear being found in AAo [5,19,20,21,22,23]. These outcomes reflect the importance of complete aortic screening, including not only the aortic root but also the proximal aorta.

### 4.2. Factors Associated with Proximal Aortic Dilatation

Studies have shown that SoV dilatation is associated with PA, right-sided aortic arch, and long duration between palliative and corrective surgery. AAo dilatation was also reported to be associated with prior palliative shunt and late corrective surgery [17,24,25]. In this study, the following factors were identified as associated with proximal aortic dilatation.

Older age at corrective surgery, especially if performed after the first year of life, was associated with proximal aortic dilatation at all levels and was correlated with higher degree of AR. Niwa et al. did not reach similar conclusions, probably due to the older age in their study sample at the time of TOF repair compared to that in our study [17]. The findings of this study support the hypothesis that volume overload across the aorta for a longer period of time may contribute to late dilatation and that early correction might have some protective effects against aortic dilatation [25,26]. 

Previous palliative shunt operation and longer duration between shunt implantation and TOF repair were associated with AAo dilatation. This finding supports previous suggestions that volume overload across the aorta attributed to shunt plays an important role in aortic root dilatation [17,24,25]. Furthermore, Anderson et. al. reported embryologically disproportionate sharing of conotruncal tissue between the aorta and pulmonary artery in favor of the aorta in TOF patients [27]. A higher degree of RVOT obstruction and lower Z-score of the pulmonary artery necessitating early palliative surgery might be embryologically associated with larger proximal aortic sizes. This hypothesis might be clearly demonstrated in patients with PA, who show a higher incidence of aortic root dilatation and AVR compared with patients that have other varieties of TOF [13].

Older age in the dilated groups during CMR emphasizes the nature of the pathology as being progressive with aging and magnifies the importance of lifelong routine follow-up. Although some studies have reported a slow rate of proximal aortic diameter progression [16], other studies have shown a higher rate of progression at the AAo level [15]. In this study, no sex predominance was found in any of the dilated groups. Right aortic sidedness was not associated with proximal aortic dilatation. This can be attributed to the exclusion of patients with PA, who have a higher prevalence of right-sided aortic arch [28].

### 4.3. Predictors of Proximal Aortic Dilatation

In the multivariate analysis, PVR and a higher degree of AR were independent predictors of AAo dilatation. No predictors of SoV dilatation were found.

#### 4.3.1. PVR

To the best of our knowledge, this study is the first to report a correlation between PVR and AAo dilatation. Three of the study subjects (15.7% of the AAo dilated group) had aortic valve and/or AAo surgery either with or after PVR. This may be due to the progressive nature of both pathologies, commonly presenting themselves late after Fallot repair. Another possible explanation is the unequal distribution of the conotruncal tissue in favor of a larger aortic root size which, again, appears as an important factor in proximal aortic dilatation [27]. Although such a relationship has not been reported in previous studies, we suggest that a severe form of unequal distribution of the conotruncal tissue—which results in a larger aortic root and a smaller pulmonary artery Z-score—may explain the correlation between proximal aortic dilatation and the higher incidence of both palliative shunt and PVR. Hypoplastic RVOT and pulmonary artery were classically addressed with palliative shunt followed by TAP implantation, which subsequently leads to severe PR and a higher rate of later PVR. In this study PVR was correlated with TAP implantation, which was performed in 57 patients during primary correction. The severity of PR was not high enough to indicate PVR in 30 patients with TAP implantation. However, further follow up of PR and proximal aortic dimensions are mandatory in those patients because of the possible future progression.

#### 4.3.2. Aortic Valve Disease

Significant AR due to proximal aortic dilatation in rTOF does not seem to be uncommon. Ordovas et al. reported 12% prevalence of an AR fraction of >15% associated with AAo and sinotubular junction dilatation in adults with rTOF [29]. Moreover, Cuypers et al. reported an increase in AR prevalence from 5% to 28% in the same study group of patients with rTOF over a follow-up period of 20 years [4], indicating the progressive nature of aortic valve dysfunction, which could be attributed to progressive proximal aortic dilatation. The sinotubular junctional dilatation associated with AAo dilatation increases tension over the free edges of valve cusps, which eventually results in reduction of the coaptation height of the aortic cusps and subsequent AR [30].

Interestingly, in our study, we found that AR severity was correlated with AAo dilatation but not with SoV dilatation. Moreover, Schäfers described frequently observed aortic root dilatation of 5 cm or more without relevant aortic regurgitation [31]. Such observations could be explained with the earlier and more common occurrence of sinotubular junctional dilatation and AR with AAo dilatation than with SoV dilatation. This hypothesis is supported by the reported decrease in the coaptation height and aortic valve performance associated with the increased ratio of sinotubular junctional diameter to aortic annular diameter [32]. Our findings support the previous recommendation of AAo screening in patients with rTOF as suggested by Cruz et al. [12]. 

### 4.4. Surgical Reinterventions

In this study, all patients with aortic surgical reintervention had significantly late TOF repair. This may reflect the effect of longstanding volume overload on the proximal aorta eventually leading to aortopathy. There was no early or late mortality after aortic surgical reintervention in our patients. However, a multicenter retrospective study that investigated the outcomes of late surgical management of aortopathy in 36 patients with rTOF reported high risk of early (14%) and late (16%) operative mortality, which was associated with larger aortic diameters [6]. This emphasizes the importance of addressing aortopathy during the planning of any future surgical reintervention, especially with PVR, which represents about one-third of re-interventions and was correlated with proximal aortic dilatation. Decision making regarding aortic surgical reintervention may be difficult and controversial in patients with borderline aortic measurements and/or moderate AR. Therefore, other important factors should also be considered. Some of these factors include the rate of progression of aortic dilatation; the severity of AR; the number of prior cardiac surgical procedures, which may increase the risk of further surgery; and the presence of family history of TAD or genetic mutation, which can accelerate the rate of aortic dilatation and dissection. Some studies have shown that both emergent [5] and elective [6] proximal aortic and/or aortic valve surgeries in patients with rTOF carry a high risk of operative mortality. Hence, addressing proximal aortic dilatation and AR in the same session as PVR may be logical. The cutoff values indicating the need for intervention require further investigation. 

### 4.5. Study Limitations

Our study was a single-center study, and its data could be influenced by survivor and migration. CMR is a part of our routine follow-up protocol, which was performed in all rTOF patients unless there is contraindication for CMR study, therefor selection bias was minimalized but cannot be excluded. Coding systems were used as a tool to identify patients with TOF and, hence, the number of included patients may be affected by inaccurate coding. Aortic angiographic CMR studies were not available before 2004, which limited the number of adequate examinations. Further, genetic syndromes were not screened in all patients. 

## 5. Conclusions

Proximal aortic dilatation and AR with subsequent AVR and/or proximal aortic surgery are common complications that occur late after TOF correction. Therefore, lifelong follow-up is mandatory, and AAo screening is recommended. CMR provides an excellent tool for follow-up with minimal risks. Aortopathy may be attributed to factors that increase the exposure of the proximal aorta to volume overload, such as late corrective surgery, palliative shunt, and residual VSD. PVR due to pulmonary valve dysfunction was one of the most common reintervention procedures performed late after total repair in this cohort of patients. PVR and a higher degree of AR were independent predictors of AAo dilatation. Aortopathy should be considered in the future management of other comorbidities, especially PR. More frequent follow-up for aortic measurements in patients who have had PVR is recommended.

## Figures and Tables

**Table 1:** Measurements of the diameter of the proximal aorta at the level of sinus of Valsalva and ascending aorta in dilated and nondilated groups.

	Nondilated SoV, *n* = 34	Dilated SoV, *n* = 43	Nondilated AAo, *n* = 58	Dilated AAo, *n* = 19
Absolute inner diameter (mm)	37.8 ± 5.0	41.3 ± 5.1	31.3 ± 4.4	40.9 ± 5.5
Inner diameter indexed to BSA (mm/m^2^)	19.3 ± 1.9	23.9 ± 2.8	16.8 ± 1.9	23.7 ± 2.8

Data are presented as mean ± standard deviation. AAo, ascending aorta; BSA, body surface area; SoV, sinus of Valsalva; mm, millimeter; m^2^, square meter; SD, standard deviation.

**Table 2:** Basic characteristics of patients with dilated sinus of Valsalva and ascending aorta.

	All Study Population	Nondilated SoV, *n* = 34	Dilated SoV, *n* = 43	*p*	Nondilated AAo, *n* = 58	Dilated AAo, *n* = 19	*p*
Male, *n* (%)	45 (58.4)	18 (52.9)	27 (62.8)	0.486	33 (56.9)	12 (63.2)	0.790
Age at CMR, y *	28.8 ± 10.6	26 ±10.07	30.8 ± 10.66	**0.024**	26.5 ± 9.6	35.5 ± 10.7	**0.002**
Age at corrective surgery, m *	52.1 ± 48.2	36.8 ± 40.6	64.1 ± 50.7	**0.004**	39.9 ± 40.6	89 ± 51.7	**<0.001**
Correction during the 1st year of life, *n* (%)	20 (26)	14 (41.2)	6 (14)	**0.009**	20 (34.5)	0 (0)	**0.002**
Palliative shunt, *n* (%)	15 (19.5)	4 (11.8)	11 (25.6)	0.156	7 (12.1)	8 (42.1)	**0.008**
Palliative shunt duration, y *	0.9 ± 2.3	0.73 ± 2.35	1.09 ± 2.29	0.156	0.6 ± 2	1.95 ± 2.89	**0.005**
Residual_VSD, *n* (%)	13 (16.9)	5 (14.7)	8 (18.6)	0.764	6 (10.3)	7 (36.8)	**0.013**
Residual VSD_duration, m *	3.6 ± 10	1.79 ± 5.35	5.05 ± 12.33	0.560	1.37 ± 4.77	10.42 ± 16.76	**0.005**
Right-sided aortic arch, *n* (%)	23 (29.9)	13 (38.2)	10 (23.3)	0.211	20 (34.5)	3 (15.8)	0.156
AR moderate to severe, *n* (%)	3 (3.9)	0 (0)	3 (7)	0.251	0 (0)	3 (15.8)	**0.013**
AR_fraction, % *	5.4 ± 10.6	2.91 ± 4.4	7.2 ± 13.32	0.370	2.29 ± 3.88	14.7 ± 17.32	**<0.001**
AVR, *n* (%)	3 (3.9)	0 (0)	3 (7)	0.251	0 (0)	3 (15.8)	**0.013**
Ascending aorta replacement, *n* (%)	2 (2.6)	0 (0)	2 (4.7)	0.500	0 (0)	2 (10.5)	0.058
TAP, *n* (%)	57 (74)	25 (73.5)	32 (74.4)	1.000	41 (70.7)	16 (84.2)	0.387
PR fraction, % *	31.4 ± 17.2	29.94 ± 14.49	32.37 ± 19.23	0.355	30.17 ± 14.38	34.73 ± 24.14	0.151
PVR, *n* (%)	28 (36.4)	8 (23.5)	20 (46.5)	0.056	14 (24.1)	14 (73.7)	**<0.001**
Arterial hypertension, *n* (%)	3 (3.9)	2 (5.9)	1 (2.3)	0.580	2 (3.4)	1 (5.3)	1.000
SBP * (mmHg)	116.4 ± 12.9	117.3 ± 13.3	115.5 ± 12.7	0.687	117.1 ± 12.9	113.8 ± 12.9	0.470
DBP * (mmHg)	71.6 ± 12.4	69.3 ± 12.2	73.4 ± 12.4	0.188	70.8 ± 11.6	74.1 ± 14.5	0.467
NYHA >II, *n* (%)	8 (10.4)	0	8 (18.6)	**0.003**	2 (3.4)	6 (31.6)	**0.002**

* Continuous data are presented as mean ± standard deviation. AAo, ascending aorta; AR, aortic regurgitation; AVR, aortic valve replacement; CMR, cardiac magnetic resonance tomography; DBP, diastolic blood pressure; m, months; NYHA, New York Heart Association classification; *n*, number; PR, pulmonary valve regurgitation; PVR, pulmonary valve replacement; SBP, systolic blood pressure; SD, standard deviation; SoV, sinus of Valsalva; TAP, transannular patch; VSD, ventricular septal defect; y, years. Significant *p* value is formatted in bold.

**Table 3:** Predictors of dilatation of sinus of Valsalva and ascending aorta by logistic regression in univariate analysis.

	Dilated SoV Group			Dilated AAo Group		
	OR	95% CI	*p*	OR	95% CI	*p*
Male	1.5	0.5–2	0.385	1.30	0.45–3.78	0.631
Age at CMR	1.05	1.00–1.10	**0.044**	1.08	1.03–1.14	**0.003**
Age at corrective surgery	1.01	1.00–1.03	**0.019**	1.02	1.01–1.03	**<0.001**
Correction during the 1st year of life	0.23	0.08–0.70	**0.009**	0.05	0.00–0.89	**0.030**
Palliative shunt	2.58	0.74–8.98	0.137	0.19	0.06–1.63	**0.047**
Palliative shunt duration	1.07	0.87–1.32	0.502	1.24	1.01–1.53	**0.043**
Residual_VSD	1.33	0.39–4.49	0.651	5.06	1.44–17.79	**0.012**
Residual VSD_duration	1.04	0.98–1.11	0.186	1.09	1.02–1.16	**0.008**
Right-sided aortic arch	0.49	0.18–1.32	0.157	2.81	0.73–10.79	0.133
AR moderate to severe	0.16	0.009–3.36	0.991	3.38	0.89–6.92	**0.013**
AR_fraction	1.06	0.99–1.13	0.106	1.20	1.07– 1.34	**0.002**
AVR	0.16	0.009–3.36	0.991	3.38	0.89–6.92	**0.003**
Ascending aorta replacement	0.24	0.01–5.11	0.992	30.2	1.35–67.11	0.992
TAP	1.05	0.38–2.92	0.930	2.21	0.57–8.59	0.252
PR fraction	1.02	0.99–1.05	0.153	1.02	0.98–1.05	0.328
PVR	2.83	1.05–7.63	**0.040**	8.80	2.69–28.78	**<0.001**
Arterial hypertension	0.38	0.03–4.39	0.439	1.56	0.13–18.18	0.725
SBP	0.99	0.96–1.03	0.561	0.98	0.94–1.02	0.352
DBP	1.03	0.99–1.07	0.160	1.02	0.98–1.07	0.319
NYHA > II	0.06	0.003–1.08	**0.030**	12.92	2.34–71.47	**0.003**

AAo, ascending aorta; AR, aortic regurgitation; AVR, aortic valve replacement; CMR, cardiac magnetic resonance tomography; CI, confidence interval; DBP, diastolic blood pressure; m, months; NYHA, New York Heart Association classification; OR, odds ratio; PR, pulmonary valve regurgitation; PVR, pulmonary valve replacement; SBP, systolic blood pressure; SoV, sinus of Valsalva; TAP, transannular patch; VSD, ventricular septal defect. Significant *p* value is formatted in bold.

**Table 4:** Predictors of dilatation sinus of Valsalva and ascending aorta by logistic regression in multivariate analysis.

	Dilated SoV Group			Dilated AAo Group		
	OR	95% CI	*p*	OR	95% CI	*p*
AR fraction	-	-	-	1.19	1.01–1.40	**0.031**
PVR	-	-	-	6.41	1.01–40.61	**0.048**
Age at corrective surgery	1.00	0.98–1.02	0.825	0.99	0.96–1.02	0.775
Correction during the 1st year of life	0.36	0.09–1.45	0.152	1.00	-	-
Age at CMR	1.01	0.94–1.09	0.694	0.93	0.81–1.06	0.310
Shunt	-	-	-	1.00	-	-
Shunt duration	-	-	-	1.42	0.91–2.21	0.115
Residual VSD	-	-	-	0.26	0.006–11.58	0.486
VSD duration	-	-	-	1.12	0.94–1.32	0.190
NYHA > II	2.10	0.84–5.23	0.109	0.91	0.49–1.68	0.779

Logistic regression analysis of dilated SoV and AAo indexed to BSA with cutoff of *p* < 0.05. AAo, ascending aorta; AR, aortic valve regurgitation; CMR, cardiac magnetic resonance imaging; CI, confidence interval; NYHA class., New York Heart Association classification; OR, odds ratio; PVR, pulmonary valve replacement; SoV, sinus of Valsalva; VSD, ventricular septal defect. Significant *p* value is formatted in bold.
